# Transcriptional Profiles of Cytokine/Chemokine Factors of Immune Cell-Homing to the Parasitic Lesions: A Comprehensive One-Year Course Study in the Liver of *E. multilocularis*-Infected Mice

**DOI:** 10.1371/journal.pone.0091638

**Published:** 2014-03-17

**Authors:** Junhua Wang, Renyong Lin, Wenbao Zhang, Liang Li, Bruno Gottstein, Oleg Blagosklonov, Guodong Lü, Chuangshan Zhang, Xiaomei Lu, Dominique A. Vuitton, Hao Wen

**Affiliations:** 1 State Key Lab Incubation Base for Xinjiang Major Diseases Research and Xinjiang Key Laboratory of Echinococcosis, First Affiliated Hospital of Xinjiang Medical University, Urumqi, Xinjiang, China; 2 Department of Nuclear Medicine, University of Franche-Comté and Jean Minjoz University Hospital, Besançon, Franche-Comté, France; 3 Institute of Parasitology, University of Bern, Bern, Switzerland; 4 WHO-Collaborating Centre for the Prevention and Treatment of Human Echinococcosis, University of Franche-Comté and University Hospital, Besançon, Franche-Comté, France; Queensland Institute of Medical Research, Australia

## Abstract

Pathogenesis of chronically developing alveolar echinococcosis (AE) is characterized by a continuous, granulomatous, periparasitic infiltration of immune cells surrounding the metacestode of *Echinococcus multilocularis (E.multilocularis)* in the affected liver. A detailed cytokine and chemokine profile analysis of the periparasitic infiltrate in the liver has, however, not yet been carried out in a comprehensive way all along the whole course of infection in *E. multilocularis* intermediate hosts. We thus assessed the hepatic gene expression profiles of 18 selected cytokine and chemokine genes using qRT-PCR in the periparasitic immune reaction and the subsequent adjacent, not directly affected, liver tissue of mice from day 2 to day 360 post intra-hepatic injection of metacestode. DNA microarray analysis was also used to get a more complete picture of the transcriptional changes occurring in the liver surrounding the parasitic lesions. Profiles of mRNA expression levels in the hepatic parasitic lesions showed that a mixed Th1/Th2 immune response, characterized by the concomitant presence of IL-12α, IFN-γ and IL-4, was established very early in the development of *E. multilocularis*. Subsequently, the profile extended to a combined tolerogenic profile associating IL-5, IL-10 and TGF-β. IL-17 was permanently expressed in the liver, mostly in the periparasitic infiltrate; this was confirmed by the increased mRNA expression of both IL-17A and IL-17F from a very early stage, with a subsequent decrease of IL-17A after this first initial rise. All measured chemokines were significantly expressed at a given stage of infection; their expression paralleled that of the corresponding Th1, Th2 or Th17 cytokines. In addition to giving a comprehensive insight in the time course of cytokines and chemokines in *E. multilocularis* lesion, this study contributes to identify new targets for possible immune therapy to minimize *E. multilocularis*-related pathology and to complement the only parasitostatic effect of benzimidazoles in AE.

## Introduction

Alveolar echinococcosis (AE) is a rare, but - if remaining untreated or treated too late- severe and fatal zoonotic helminthic disease, predominantly caused not only by the direct hepatic damage which follows the continuous tumor-like proliferation of the larval stage (metacestode) of *Echinococcus multilocularis (E.multilocularis)*, but also indirectly by the intense local granulomatous immune response which surrounds the parasitic tissue [1]. Granuloma, extensive fibrosis, and necrosis are actually the characteristic pathological findings in *E. multilocularis* infection. The lesions, composed both of the multiple vesicle-forming metacestode and of cells homing from lymphoid organs and permanently settling around the metacestode, behave like a slow-growing liver cancer, progressively invading the liver, then the neighboring tissues and also metastazing to other organs [2]. Pathological changes in AE are associated with an intense infiltration by immune cells, i.e. macrophages of various functional types, including the so-called “epithelioid cells” and “giant cells”, typical of granulomas [3] and T lymphocytes. CD4^+^ T lymphocytes are present from the early stage of parasite growth and CD8^+^ T lymphocytes are known to home to the periparasitic infiltrate secondarily and to be associated with parasite tolerance and severity of the disease [1], [2], [3], [4]. Non-immune cells such as fibroblasts and myofibroblasts which are crucial for the development of fibrosis are also attracted by the host's immune response around the parasite.

It has been shown that *E. multilocularis* infection induced numerous pathways of the immune response; the involvement of individual cytokines has been rather extensively studied within the past 2 decades both in humans and in experimental rodents [1]. In the immune-competent but susceptible host, *E. multilocularis* induces skewed Th2-responses [5]. In chronic AE, Th2-dominated immunity is associated with increased susceptibility to disease, while Th1 cell activation induces a rather protective immunity which involves IFN-α [6] and IL-12 [7] as initiating cytokines, and IFN-γ [8] and TNF-α [9], [10] as effector cytokines. During the course of *E. multilocularis* infection, as studied in mice, an initial acute stage Th1 response gradually switches to an increasingly dominating Th2 response; the thus mostly mixed Th1/Th2 profile of the chronic stage is associated with the expression of pro-inflammatory cytokines in the granuloma [11], [12]. Th2 cytokines down-modulate the Th1 response which nevertheless decreasingly persists all along the infection until the late pre-mortem immune-suppressed stage of AE [11]. The metacestode actively achieves a tolerance status through the induction of regulatory cytokines, such as IL-10 and TGF-β [11]. However, this bulk of information has mostly been obtained from studies on peripheral blood mononuclear cells (in humans), and on spleen and lymph node cells in the experimental model [5], [13], [14]. In addition, nothing was known until very recently about role of IL-17 and Th17 cells [13], [14] during *E. multilocularis* infection. Only two studies have given some insight into chemokine [15], [16] and IL-17 [17] involvement in *E. multilocularis* infection, respectively; and this was done only in AE patients, and never in the infected liver tissue; the actual involvement of IL-17 and chemokines in the lesions is thus unknown. The time course of IL-17 expression is also unknown since human AE is usually discovered late, i.e. years after *E. multilocularis* infection of the patients, and findings in humans reflect only the late chronic stage of infection. Studies in the experimental mouse model are therefore necessary to dissect the various stages of *E. multilocularis* infection regarding the host's immune response.

In the present report, our objectives were to 1) give a comprehensive appraisal of the various components, especially cytokines and chemokines, involved in immune cell homing around the *E. multilocularis* metacestode, at the various successive stages of disease, i.e. early, middle and late stages as defined previously [18], [19], and 2) to study the parasite and the host immune response in their usual context, the liver, in the experimental mouse model of hepatic secondary infection. Eighteen key-cytokines and -chemokines were measured both in the lesion, including the periparasitic infiltrate, and in the surrounding liver, close to the lesions, using qRT-PCR. To get a more complete picture of the influence of the parasite-induced host's immune response on the host's liver, a microarray technique was also used to study the surrounding liver tissue.

## Results

### Hepatic histopathology during *E. multilocularis*-infection

From day 2 to day 360 post-infection (p.i.) with *E. multilocularis*, the hepatic parasitic lesions showed the various morphological patterns specific to the different stages of murine AE, as described in a previous study using the same experimental mice (data not shown) [18], [19]. According to previous reports on the course of *E. multilocularis* secondary infection in experimental susceptible mice [18], [19], the 3 main stages were defined as follows: early stage, from infection to day 60; middle stage from day 60 to day 180; and late stage from day 180 to day 360.

### Innate immunity and pro-inflammatory cytokines

**Figure 1:**
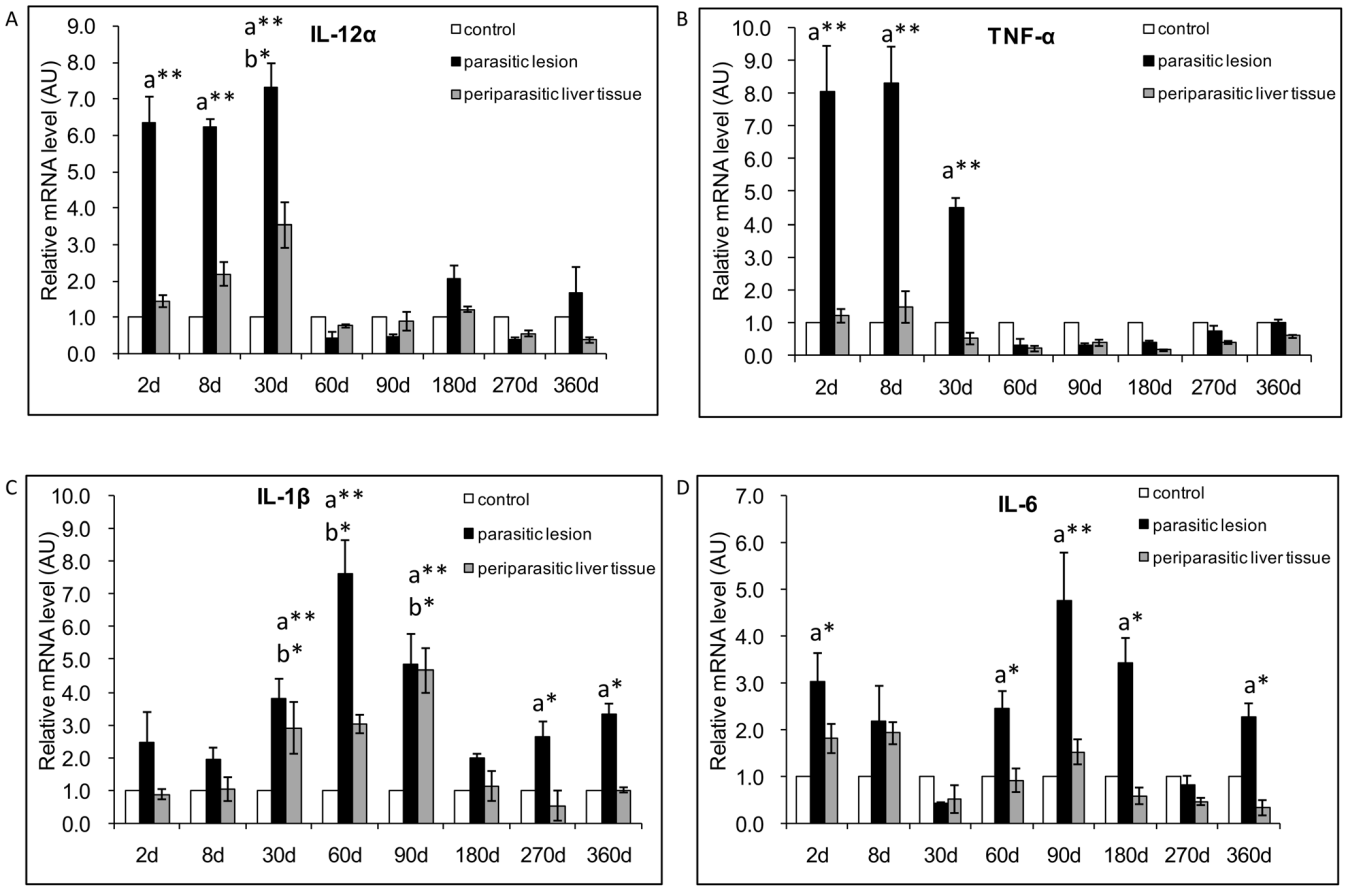
Course of IL-12α and pro-inflammatory cytokine gene expression in the liver of mice during *E. multilocularis* infection (measured by qRT-PCR). (A) IL12α, (B) TNF-α, (C) IL-1B, (D) IL-6. a: ‘Parasitic lesion’ versus ‘Control’; b: ‘Periparasitic liver tissue’ versus ‘Control’. *P<0.05; **P<0.01. ‘Control’, non-infected mice; ‘Parasitic lesion’: *E. multilocularis* metacestode and surrounding immune infiltrate; ‘Periparasitic liver tissue: liver parenchyma close to the *E. multilocularis* lesion, but excluding macroscopically visible liver tissue alterations. AU: arbitrary units.

In *E. multilocularis* ‘parasitic lesions’ (i.e. including adjacent infiltrates, as defined in the Materials and Methods section), qRT-PCR showed that IL-12α mRNA expression was 6.3-fold higher at as early as day 2 p.i. than in control mice (Figure 1A). There was a significant difference between *E. multilocularis*-infected mice and control mice, at the early stage of infection, at time points of 2-, 8- and 30-day p.i. (*P*<0.05). In the ‘periparasitic liver tissue’ (i.e. liver parenchyma close to the lesions, as defined in the Materials and Methods section), IL-12α mRNA expression was also higher than in control livers from day 8 to day 30 p.i.. There was a significant difference at 30-days p.i. (*P*<0.05). Changes in IL-12α mRNA expression with time are shown in Figure 1A.

In *E. multilocularis* lesions, qRT-PCR showed that TNF-α mRNA expression was increased at the early stage of infection, especially at days 2 and 8 p.i.; it remained high at 30 days p.i. but decreased subsequently (Figure 1B). There was a significant difference between *E. multilocularis* infected mice and control mice, at the time points of 2-, 8- and 30-day p.i. (*P*<0.05). In the periparasitic liver tissue, TNF-α mRNA expression did not change from day 2 to day 360 (Figure 1B). In the lesions, there was an increase in IL-1β mRNA expression all over the infection course, from day 2 to day 360 p.i., with a peak at 60 days p.i.. IL-1β mRNA expression was 2.5-fold higher at day 2 and 7.6-fold higher at day 60 (Figure 1C), when compared to control mice. There was a significant difference between *E. multilocularis* infected mice and control mice, at the time points of 30-, 60-, 90-, 270- and 360-days p.i. (*P*<0.05). In the liver tissue, IL-1β mRNA expression increased later, from 2.9-fold at day 30 to 4.7-fold at day 90 (Figure 1C), and was at its maximum at the middle stage of infection. There was a significant difference at the time points of 30-, 60- and 90-days p.i. (*P*<0.05). In the lesions, IL-6 mRNA expression was markedly increased as early as 2 days; then it relatively decreased at day 30 p.i., then re-increased very significantly from day 90 p.i. (4.8-fold) (Figure 1D). There was a significant difference between *E. multilocularis* infected mice and control groups, at the time points of 2-, 60-, 90-, 180- and 360-days p.i. (*P*<0.05). In the liver, IL-6 mRNA expression increased at the very early stage of infection, 1.8-fold at day 2 and1.9-fold at day 8 (Figure 1D); it returned back to normal at day 30, and re-increased from day 60 to day 90, then a high level was maintained until day 360.

### Th1 cytokines and related chemokines

#### Th1 cytokines

**Figure 2:**
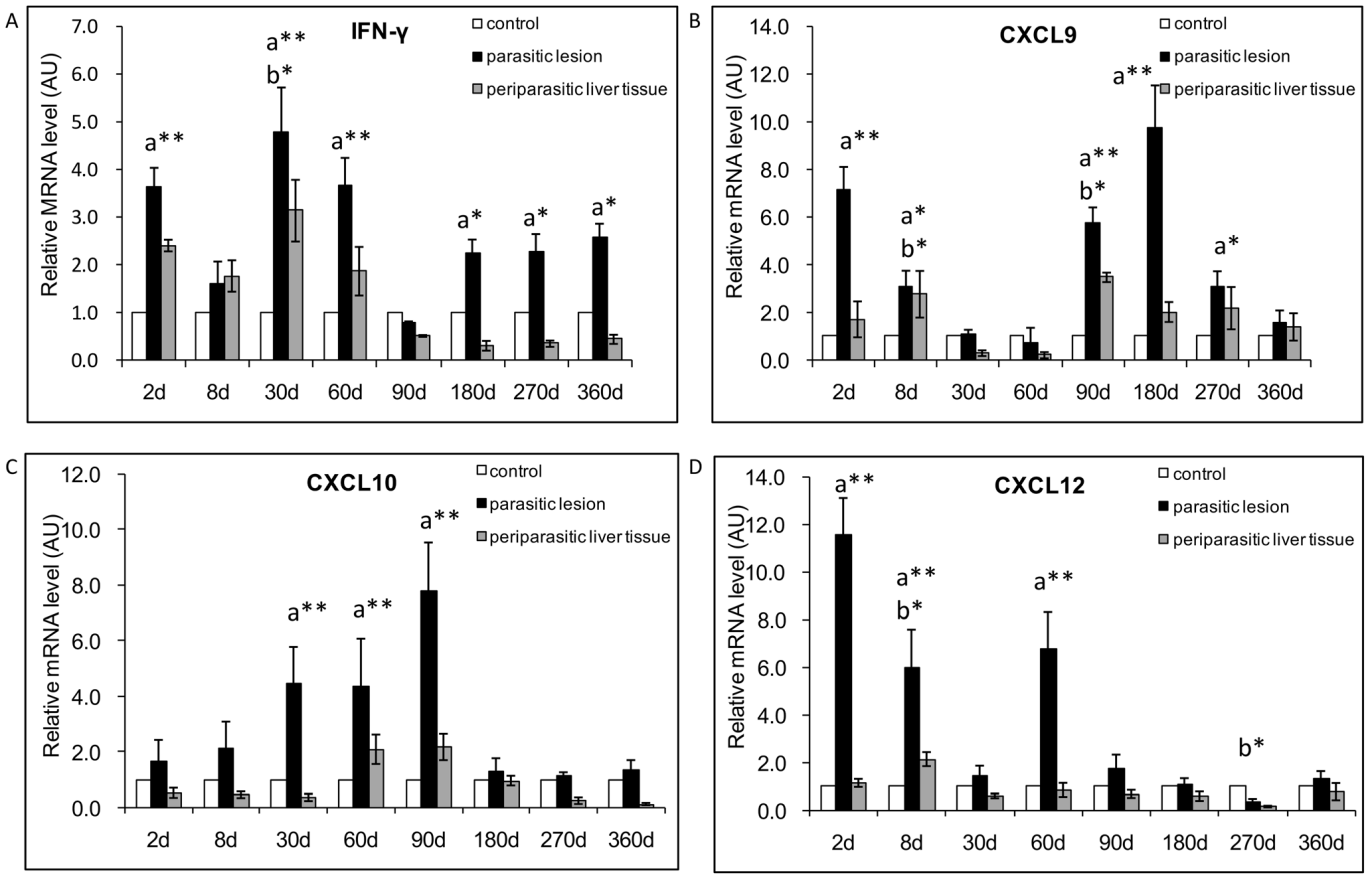
Course of Th1-cytokine and related chemokine gene expression in the liver of mice during *E. multilocularis* infection (measured by qRT-PCR). (A) IFN-γ, (B) CXCL9, (C) CXCL10, (D) CXCL12. a: ‘Parasitic lesion’ versus ‘Control’; b: ‘Periparasitic liver tissue’ versus ‘Control’. *P<0.05; **P<0.01. ‘Control’, non-infected mice; ‘Parasitic lesion’: *E. multilocularis* metacestode and surrounding immune infiltrate; ‘Periparasitic liver tissue: liver parenchyma close to the *E. multilocularis* lesion, but excluding macroscopically visible liver tissue alterations. AU: arbitrary units.

In the lesions, an increase in IFN-γ mRNA expression was observed from day 2 to day 360 p.i., with a peak at 30 days p.i.. Except for an apparent decrease at day 8, IFN-γ mRNA-expression was especially increased at the early stage of infection, from 3.6-fold at day 2 to 4.8-fold at day 30 (Figure 2A). There was a significant difference between *E. multilocularis* infected mice and control mice, at the time points of 2-, 30-, and 60-day p.i., but also at the latest stage, 360- day p.i. (*P*<0.05). In the liver, IFN-γ mRNA expression was increased from 2.4-fold at day 2 to 3.1-fold at day 30 (Figure 2A), but became abrogated at the late stage of infection, from 0.5- fold at day 90 to 0.4 at day 360, compared to control mice. There was a significant difference at the time point of 30-day p.i. (*P*<0.05).

#### Th1-related chemokines

Expression of CXCL9 mRNA was observed from day 2 to day 360 p.i.. In the lesions, CXCL9 mRNA expression was increased from day 90 to day 360, with a peak of 9.75-fold at day 180 (Figure 2B), compared to control mice. There was a significant difference between *E. multilocularis*-infected mice and control mice, at the time points of 2-, 8-, 90-, 180- and 270-days p.i., i.e. at the late stage of infection (*P*<0.05). In the liver, CXCL9 mRNA expression was increased by 1.72-fold at day 2 and 2.78-fold at day 8 (Figure 2B); it was decreased by 0.30- fold at day 30 and by 0.21-fold at day 60, then expression re-increased by 3.5- fold at day 90 compared to control mice. There was a significant difference at the time points of 8- and 90-day p.i. (*P*<0.05). In the lesions of *E. multilocularis*-infected mice, CXCL10 mRNA expression was increased by 1.6-fold at day 2; then levels progressively increased to a peak (7.8-fold the levels in control mice) at day 90 p.i. (Figure 2C). There was a significant difference between *E. multilocularis* infected mice and control mice, at the time points of 30-, 60- and 90-days p.i. (*P*<0.05), i.e. at the middle stage of infection. In the liver, CXCL10 mRNA expression was increased at day 60 (2.1-fold) and at day 90 (2.2-fold) (Figure 2C), and was lower both at the early stage and the late stage of infection when compared to control mice. Expression of the mRNA of CXCL12, a chemotactic factor for lymphocytes, was observed from day 2 to day 360 p.i.. In the lesions, CXCL12 mRNA expression was markedly increased as early as day 2 post-infection, when it reached a peak (11.6-fold); it remained elevated until day 60(Figure 2D). There was a significant difference between *E. multilocularis* infected mice and control groups, at the time points of 2-, 8- and 60-days p.i. (*P*<0.05). In the liver, CXCL12 mRNA expression was increased early, from 1.1-fold at day 2 to 2.1-fold at day 8 (Figure 2D), and was lower than that observed in control mice at the late stage, from day 90 to day 360. There was a significant difference at the time points of 8- and 270-days p.i. (*P*<0.05).

### Th2 cytokines and related chemokines

#### Th2 cytokines

**Figure 3:**
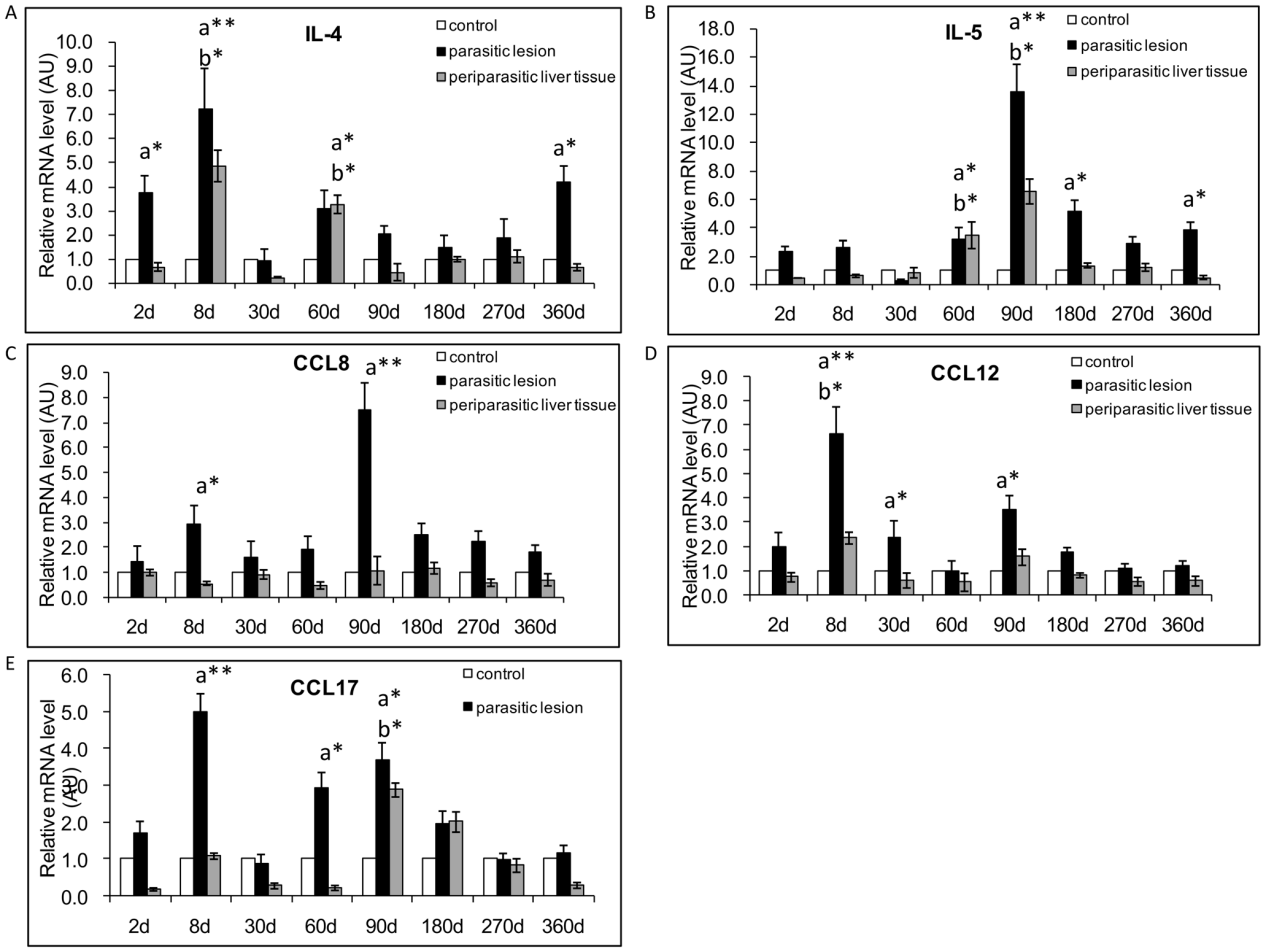
Course of Th2-cytokine and related chemokine gene expression in the liver of mice during *E. multilocularis* infection (measured by q RT-PCR). (A) IL-4, (B) IL-5, (C) CCL8, (D) CCL12, (E) CCL17. a: ‘Parasitic lesion’ versus ‘Control’; b: ‘Periparasitic Liver tissue’ versus ‘Control’. *P<0.05; **P<0.01. ‘Control’, non-infected mice; ‘Parasitic lesion’: *E. multilocularis* metacestode and surrounding immune infiltrate; ‘Periparasitic liver tissue: liver parenchyma close to the *E. multilocularis* lesion, but excluding macroscopically visible liver tissue alterations. AU: arbitrary units.

In *E. multilocularis* lesions, IL-4 mRNA expression followed a biphasic curve: it was increased early (3.8-fold at day 2), and was significantly different from that observed in control mice at 2 and 8 days; but it relatively decreased at 30 p.i.; it then re-increased and was still elevated at the late stage [4.2-fold at day 360; significantly different from control mice (*P*<0.05). (Figure 3A)]. In the liver, IL-4 mRNA expression was increased compared to control mice [4.8-fold at day 8 and 3.2-fold at day 60, significantly different from control mice (*P*<0.05) (Figure 3A)]. In *E. multilocularis* lesions, IL-5 mRNA expression was present from the early stage (2.3-fold at day 2); however (Figure 3B), there was a peak of 13.6-fold at day 90, and a significant difference between *E. multilocularis* infected mice and control mice, all over the middle and late stages of infection, at the time points of 60-, 90-, 180- and 360-days p.i. (*P*<0.05). In the liver, IL-5 mRNA expression was also markedly increased at the middle stage of infection: 3.5-fold at day 60 and 6.54-fold at day 90 (Figure 3B). There was a significant difference at the time points of 60- and 90-days p.i. (*P*<0.05).

#### Th2-related chemokines

In the lesions, mRNA expression of CCL8, chemotactic for and activator of various immune cell types, including mast cells, eosinophils and basophils, monocytes, T cells, and NK cells [22], was increased from day 2 to day 360 p.i., with a peak at day 90 (Figure 3C). There was a significant difference between *E. multilocularis* infected mice and control mice, at the very early and at the middle stage of infection, at the time points of 8- and 90-days p.i. (*P*<0.05). In the liver, there was no difference in CCL8 mRNA expression from day 2 to day 360 (Figure 3C) between infected and control mice. In the lesions, mRNA expression of CCL12, another Th2-related chemokine, which attracts eosinophils, monocytes and lymphocytes [25], increased early, from 2.0-fold at day 2 to 6.6-fold at day 8 p.i. when it became significantly different from control mice (Figure 3D); levels were also elevated at day 90 p.i. (3.5-fold; also significantly different from control mice). In the liver, CCL12 mRNA expression did not change from day 2 to day 360 (Figure 3D), compared to control mice. mRNA expression of CCL17, which induces T-cell chemotaxis and elicits its effects by interacting with the chemokine receptor CCR4, was observed in the lesions (1.7- fold increase at day 2 and 2.0-fold at day 180 p.i.), (Figure 3E). There was a significant difference between *E. multilocularis* infected mice and control groups, at the time points of 8-, 60- and 90-days p.i., when its expression peaked at 3.7 fold (*P*<0.05). A slight decrease in CCL17 mRNA expression was observed at day 30 p.i., concomitant to the slight decrease also observed for the Th2-related cytokines IL-4 and IL-5. In the liver, CCL17 mRNA expression was higher than in control mice from day 2 to day 180 (Figure 3E). There was a significant difference at 90-days p.i. between infected and control mice (*P*<0.05).

### Th17 cytokines

#### IL-17 and its isotypes

**Figure 4:**
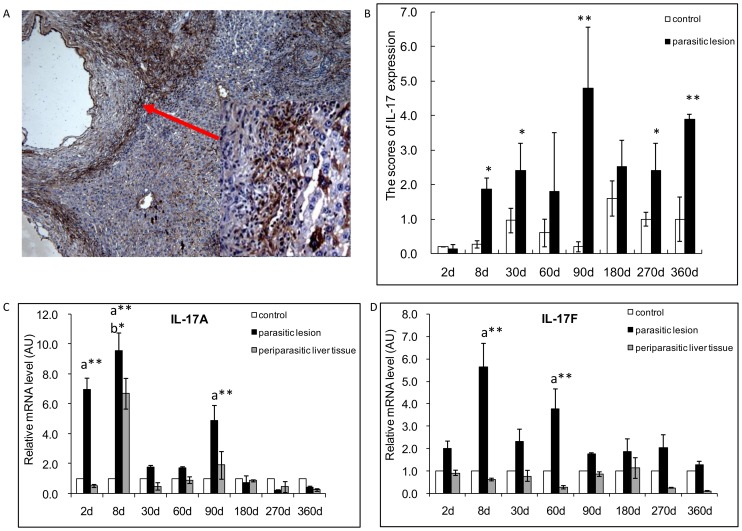
Th17-cytokine cytokine expression in the liver of mice during *E. multilocularis* infection. (A). Expression of IL-17 at day 90. IL-17 was present in most of the infiltrating lymphocytes of areas with inflammatory granulomas, in the cytoplasm of hepatocytes, endothelial cells of the hepatic sinusoids and fibroblasts (*arrow* indicates the area also shown at high magnification). (B). Expression scores of IL-17, calculated from quantitative analysis of the histo-immunostaining using both staining intensity and the percentage of cells stained at a specific range of intensities (see Materials and Methods section). (C). Course of IL-17A, and (D) of IL-17F gene expression in the liver of mice during *E. multilocularis* infection (measured by q RT-PCR). a: ‘Parasitic lesion’ versus ‘Control’; b: ‘Periparasitic liver tissue’versus ‘Control’. *P<0.05; **P<0.01. ‘Control’, non-infected mice; ‘Parasitic lesion’: *E. multilocularis* metacestode and surrounding immune infiltrate; ‘Periparasitic liver tissue: liver parenchyma close to the *E. multilocularis* lesion, but excluding macroscopically visible liver tissue alterations. AU: arbitrary units.

In the periparasitic infiltrate area, IL-17, disclosed by immunostaining (Figure 4A), was observed in most lymphocytes and macrophages in the periparasitic infiltrate, as well as in fibroblasts, and endothelial cells in hepatic sinusoids, especially around the granulomas, and in infiltrating immune cells of portal spaces, from day 8 to day 360 p.i.. IL-17 positive scores ranged from 0.13 to 4.80 and reached the peak point at day 90p.i. (Figure 4B). In the liver close to the parasite lesions, moderate IL-17 expression was observed; there was a significant difference between AE-infected and sham-injected mice at day-8, -30, -90, 270 and 360p.i..

In *E. multilocularis* lesions, IL-17A mRNA expression was increased at the very early stage of infection, by 6.9-fold at day 2 and by 9.6-fold at day 8 p.i. (Figure 4C), and decreased at the late stage, from day 180 to day 360 p.i.. There was a significant difference between *E. multilocularis* infected mice and control groups, at the time points of 2-, 8- and 90-days p.i. (*P*<0.05). In the liver, IL-17A mRNA expression was also increased at the very early stage: 6.7-fold at day 8; at this time point, the difference was significant (Figure 4C) (*P*<0.05). In the lesion, IL-17F mRNA expression was present all over the infection course, from day 2 to day 360 p.i. (Figure 4D), with a peak of 5.63-fold at day 8 compared to control mice. There was a significant difference between *E. multilocularis* infected and control mice, at the time points of 8- and 60-days p.i. (*P*<0.05). At the late stage, despite an apparent increase, compared to control mice, the difference was not significant. In the liver, IL-17F mRNA expression did not change significantly from day 2 to day 360 (Figure 4D).

### Treg-related nuclear transcriptional factor and cytokines

#### Treg related nuclear transcriptional factor (Foxp3)

**Figure 5:**
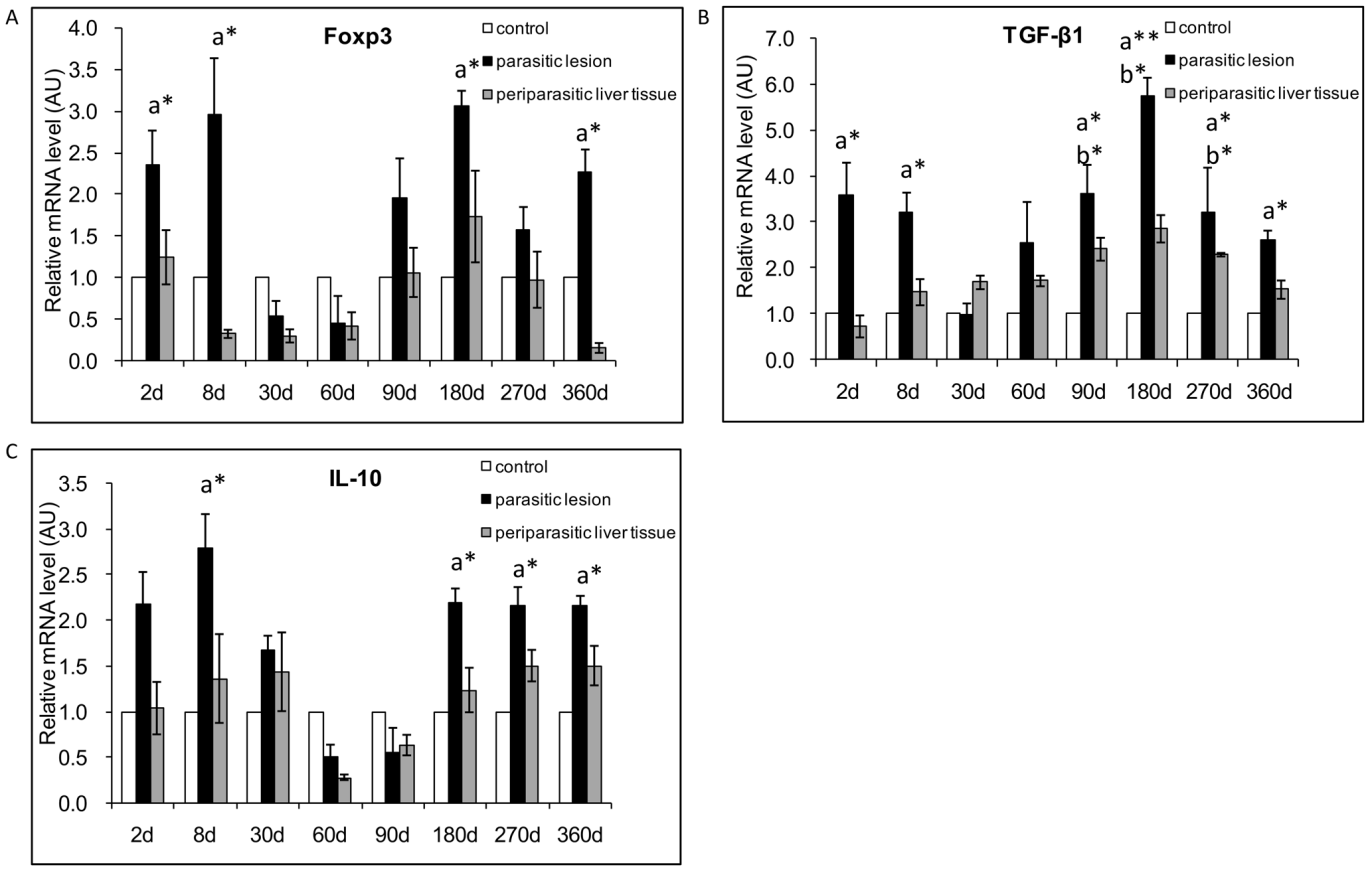
Course of Treg transcription factor and Treg-cytokine gene expression in the liver of mice during *E. multilocularis* infection (measured by q RT-PCR). (A) Foxp3, (B) TGF-β1, (C) IL-10. a: ‘Parasitic lesion’ versus ‘Control’; b: ‘Periparasitic liver tissue’ versus ‘Control’. *P<0.05; **P<0.01. ‘Control’, non-infected mice; ‘Parasitic lesion’: *E. multilocularis* metacestode and surrounding immune infiltrate; ‘Periparasitic liver tissue: liver parenchyma close to the *E. multilocularis* lesion, but excluding macroscopically visible liver tissue alterations. AU: arbitrary units.

In *E. multilocularis* lesions, Foxp3 mRNA expression was increased by 2.4-fold at day 2 and by 3.0-fold at day 8 p.i. (Figure 5A); it then decreased from day 30 to day 60 p.i., and re-increased, from 1.9-fold at day 90 to 2.3-fold at day 360 p.i., with a peak of 3.1-fold at day 180, at the late stage of infection (Figure 5A), thus following a biphasic curve in the course of infection. There was a significant difference between *E. multilocularis* infected mice and control mice, at the time points of 2-, 8-, 180- and 360-days p.i. (*P*<0.05). In the liver, there was no significant change in Foxp3 mRNA expression (Figure 5A).

#### Treg-related cytokines

In *E. multilocularis* lesions, TGF-β1 mRNA expression also followed a biphasic curve, with a decrease at days 30 and 60 p.i.; it was increased by 3.6-fold at day 2 and 3.2-fold at day 270 p.i. (Figure 5B) with a peak of 5.7- fold at day 180 (Figure 5B). There was a significant difference between *E. multilocularis* infected mice and control mice, at the early and late stages of infection, at time points of 2-, 8-, 90-, 180-, 270- and 360-day p.i. (*P*<0.05). In the liver, TGF-β1 mRNA expression was also increased from day 8 to day 360 p.i., with a peak at day 180 p.i.. Conversely to the expression of TGF-β1 mRNA in the lesions, in the liver, TGF-β1 mRNA was significantly elevated at the middle and late stages, at the time points of 90-, 180- and 270-days p.i. (*P*<0.05). In *E. multilocularis* lesions, IL-10 mRNA expression was also biphasic, with a significant increase at the early and late stages of infection, but not at its middle stage (Figure 5C). There was a significant difference between *E. multilocularis* infected mice and control mice, at the time point of 8-day, then at 180-, 270- and 360-days p.i. (*P*<0.05). In the liver, IL-10 mRNA expression did not change from day 2 to day 360 (Figure 5C) compared to control mice.

### Immune response and inflammation gene expression in the liver of *E. multilocularis* infected mice

To further give a comprehensive picture of the immune response-related changes in the adjacent liver during *E. multilocularis* infection, and especially detect hyper-expression of the genes of cytokine/chemokine receptors, cDNA microarray technology was used. The individual genes associated with the gene ontology biological process “immune response”, and “pathogen response” assessed at different time periods of infection, i.e. 30, 60, 90, 180 days p.i., are presented in Table 1. We used Gene Ontology (GO; www.geneontology.org) analysis which clusters the genes associated with immune response/defense (n = 59) into functional subgroups including macrophages, APCs, chemokines and chemokine receptors, lymphocytes, B-cells and eosinophils.

**Table 1:** Gene ontology category: immune response and inflammatory response.

GeneBank accession number	Gene Symbol	Name	30 days	60 days	90 days	180 days
76074	5830443L24Rik	RIKEN CDNA 5830443L24 gene	*	*	2.84	*
11699	Ambp	Alpha 1 microglobulin/bikunin (Ambp)	−2.35	*	*	*
56298	Arl6ip2	ADP-ribosylation factor-like 6 interacting protein 2	*	*	2.05	*
236573	BC057170	cDNA sequence BC057170	*	*	3.41	*
12260	C1qb	Complement component 1, q subcomponent, beta polypeptide	*	*	*	2.12
12262	C1qc	Complement component 1, q subcomponent, C chain	*	*	*	3.58
12279	C1qg	Complement C1q subcomponent, C chainprecursor	*	*	*	2.18
625018	C4b	Complement component 4B (Childo blood group)	*	3.09	*	*
230558	C8a	Complement component 8, alpha polypeptide	*	*	*	3.98
20304	Cc15	Chemokine (C-C motif) ligand 5	2.35	*	*	*
20307	Ccl8	chemokine (C-C motif) ligand 8	29.58	*	*	*
20293	Ccl12	chemokine (C-C motif) ligand 12	5.64	*	*	*
20295	Ccl17	chemokine (C-C motif) ligand 17	3.36	*	*	*
93671	Cd163	CD163 antigen	*	*	*	2.56
12500	Cd3d	CD3 antigen, delta polypeptide (Cd3d)	2.14	*	*	*
23833	Cd52	CD52 antigen	*	*	*	2.48
12516	Cd7	CD7 antigen (Cd7)	2.19	*	*	*
12525	Cd8a	CD8 antigen, alpha chain (Cd8a)	4.41	*	*	*
12526	Cd8b1	CD8 antigen, beta chain 1	3.13	*	*	*
12628	Cfhr1	Complement factor H-related 1	*	*	2.66	*
18636	Cfp	Complement factor properdin	*	*	*	2.19
17474	Clec4d	C-type lectin domain family 4, member d (Clec4d)	3.97	*	*	*
56619	Clec4e	C-type lectin domain family 4, member e (Clec4e)	5.08	*	*	*
17329	Cxcl9	Chemokine (C-X-C motif) ligand 9	*	*	*	2.81
20315	Cxcl12	chemokine (C-X-C motif) ligand 12	*	*	*	−2.21
14131	Fcgr3	Fc receptor, IgG, low affinity III	*	*	*	2.73
55932	Gbp3	Guanylate nucleotide binding protein 3	*	*	2.15	*
15139	Hc	Hemolytic complement	*	*	*	2.03
15439	Hp	Haptoglobin	*	*	*	2.75
17082	I11r1	Interleukin 1 receptor-like 1 (Il1rl1), transcript variant 2	2.92	*	*	*
16197	I17r	Interleukin 7 receptor (Il7r)	2.25	*	*	*
16164	Il13ra1	interleukin 13 receptor, alpha 1	*	*	2.39	*
16172	Il17r	interleukin 17 receptor D	*	*	*	2.9
26388	Ifi202b	Interferon activated gene 202	*	*	2.88	*
15950	Ifi203	Interferon activated gene 203	*	*	2.13	*
15951	Ifi204	Interferon activated gene 204	*	*	2.47	*
16010	Igfbp4	Insulin-like growth factor bindingprotein 4	*	2.13	*	*
16797	Lat	Linker for activation of T cells (Lat)	2.12	*	*	*
17395	Mmp9	Matrix metallopeptidase 9 (Mmp9)	2.74	*	*	*
17312	Mgl1	macrophage galactose N-acetyl-galactosamine specific lectin 1	2.56	*	*	*
216864	Mgl2	macrophage galactose N-acetyl-galactosamine specific lectin 2	4.64	*	*	*
100702	Mpa2l	macrophage activation 2 like	*	−2.33	3.99	*
20288	Msr1	macrophage scavenger receptor 1	*	2.25	*	*
80891	Msr2	macrophage scavenger receptor 2	*	*	2.11	*
18405	Orm1	Orosomucoid 1	*	*	*	2.61
18406	Orm2	Orosomucoid 2	*	2.67	*	8.94
18514	Pbx1	Pre B-cell leukemia transcription factor 1	*	*	2.19	*
233489	Picalm	Phosphatidylinositol binding clathrin assembly protein	*	*	2.01	*
27226	Pla2g7	Phospholipase A2, group VII (platelet-activating factor acetylhydrolase, plasma)	*	*	*	2.57
18761	Prkcq	Protein kinase C, theta	*	*	−3.22	*
20208	Saa1	Serum amyloid A 1	*	*	*	11.63
20210	Saa3	Serum amyloid A 3	*	*	*	9.69
20211	Saa4	Serum amyloid A 4	*	*	*	2.32
20714	Serpina3k	Serine (or cysteine) peptidase inhibitor, clade A, member 3K (Serpina3k)	−3.38	*	*	*
20716	Serpina3n	Serine (or cysteine) peptidase inhibitor, clade A, member 3N	*	*	*	3.12
20750	Spp1	Secreted phosphoprotein 1	*	*	3.54	*
192187	Stab1	Stabilin 1	*	*	*	2.04
21822	Tgtp	T-cell specific GTPase	*	*	*	2.79
107568	Wwp1	WW domain containing E3 ubiquitin protein ligase 1	*	*	2.37	*

Genes with up- or down-regulated transcriptions in the liver of *Echinococcus multilocularis* (*E.multilocularis*)-infected BALB/c mice are shown in comparison with non-infected sham-injected control animals (fold increase/decrease).

More precisely, at 30 days p.i., several biological processes relating to an active infection, as defined by GO cluster classification, were involved, including genes mostly associated with the response to external stimuli, response to wounding, immune response, response to stress, chemokine activity, defense response, MHC-related functions and inflammatory response. While several chemokine genes were found activated in the liver of AE mice by qRT-PCR, microarray analysis did not show any up-regulation of cytokine genes. Among genes of cytokine receptors, only those for IL-1 (IL-R1 like) and IL-7 (2.92 and 2.25 fold respectively) were up-regulated at day 30 (Table 1). Among genes encoding for chemokines, CCL5 (RANTES), a Th17-related chemokine that up-regulates IL-12 and IFN-γ, and is involved in Th1 cell-migration [16], was up-regulated 2.35-fold at day 30. Th2-related CCL8, CCL12 and CCL17 were up-regulated 29.58-fold, 5.64-fold and 3.36-fold at day 30, respectively. Among genes related to macrophage function, MGL1 and MGL2 (C-type macrophage galactose-type lectins) were up-regulated 2.56- and 4.64-fold respectively, compared to control mice.

At 60 days p.i., genes involved in the response to stress, response to external stimulus and response to biotic stimuli were added. There were few changes in the immune response gene expression, except for MPA2L (macrophage activation 2-like), which was down-regulated 2.33-fold and C4b (Complement component 4B), which was up-regulated 3.09-fold, respectively.

At 90 days p.i., among genes encoding for cytokine receptors, IL-13 Rα1 was up-regulated 2.39-fold (Table 1). Among the interferon-activated genes, Ifi202b, Ifi203 and Ifi204 were up-regulated 2.88-, 2.13-, and 2.47-fold, respectively. Among genes encoding for macrophage functions, MSR1 (macrophage type-I class-A scavenger receptors) and MPA2L (macrophage activation 2-like) were up-regulated 2.11- and 3.99-fold, respectively, when compared to control mice.

At 180 days p.i., hyper-expression of genes of the inflammatory response, response to stress, and response to external stimuli was maintained, and genes of antigen processing and presentation, complement activity and antigen processing via MHC class II were also hyper-expressed. Among genes of cytokine receptors, IL-17R was up-regulated 2.90-fold (Table 1). Among genes encoding for chemokines, CXCL9 was up-regulated 3.81-fold at day 180, and CXCL12 was down-regulated 2.11-fold at day 180.

### Correlations between mRNA levels of the various cytokines over the course of infection

Spearman correlation coefficients indicated a significant positive correlation between TGF-β1 mRNA expression in *E. multilocularis* ‘parasitic lesion’, and that of Foxp3 (r = 0.719, *P* = 0.045), IL-10 (r = 0.761, *P* = 0.028) and CXCL9 (r = 0.946, *P*<0.01), but a significant negative correlation with IFN-γ (r = −0.743, *P* = 0.035) (Table 2); it also showed a significant positive correlation between Foxp3 expression in *E. multilocularis* ‘parasitic lesion’, as measured by qRT-PCR, and IL-10 (r = 0.761, P = 0.028) and TNF-α (r = 0.742, P = 0.035), but a significant negative correlation with IL-1β (r = -0.754, *P* = 0.033) (Table 3). There was a significant positive correlation between IL-17A expression in *E. multilocularis* ‘parasitic lesion’, as measured by qRT-PCR, and CCL12 (r = 0.833, *P* = 0.011), CCL17 (r = 0.733, *P* = 0.039), IL-4 (r = 0.710, *P* = 0.049) and TNF-α (r = 0.804, *P* = 0.016) (Table 4); there was also a significant positive correlation between IL-17F mRNA expression in *E. multilocularis* ‘parasitic lesion’ and CCL12 (r = 0.708, *P* = 0.049) and CCL17 (r = 0.749, *P* = 0.032)(Table 4). TNF-α mRNA expression in *E. multilocularis* ‘parasitic lesion’ was also significantly correlated to IL-12α (r = 0.888, *P* = 0.033) (Table 5).

**Table 2:** Correlations between mRNA of TGF-β1 and Foxp3, IL-10, IFN-γ and CXCL9.

		Foxp3	IL-10	IFN-γ	CXCL9
TGF-β1	Spearman's rho	0.719*	0.761**	−0.743*	0.946**
	Sig.	0.045	0.028	0.035	0.000
	N	8	8	8	8

Note:

* *P*<0.05,

** *P*<0.01.

**Table 3:** Correlations between mRNA of Foxp3 and TGF-β1, IL-10, IL-1β and TNF-α.

		TGF-β1	IL-10	IL-1β	TNF-α
Foxp3	Spearman's rho	0.719*	0.761**	−0.754*	0.742*
	Sig.	0.045	0.028	0.033	0.035
	N	8	8	8	8

Note:

* *P*<0.05,

** *P*<0.01.

**Table 4:** Correlations between mRNA of IL-17 and CCL12, CCL17, IL-4, and TNF-α.

		CCL12	CCL17	IL-4	TNF-α
IL-17A	Spearman's rho	0.833*	0.733*	0.710*	0.804*
	Sig.	0.011	0.039	0.049	0.016
	N	8	8	8	8
IL-17F	Spearman's rho	0.708*	0.749*	0.695	0.497
	Sig.	0.049	0.032	0.056	0.210
	N	8	8	8	8

Note:

* *P*<0.05,

** *P*<0.01.

**Table 5:** Correlations between mRNA of TNF-α and IL-12α, as well as IL-17A.

		IL-12α	IL-17A
TNF-α	Spearman's rho	0.888**	0.804*
	Sig.	0.003	0.016
	N	8	8

Note:

* *P*<0.05,

** *P*<0.01.

## Discussion

**Figure 6:**
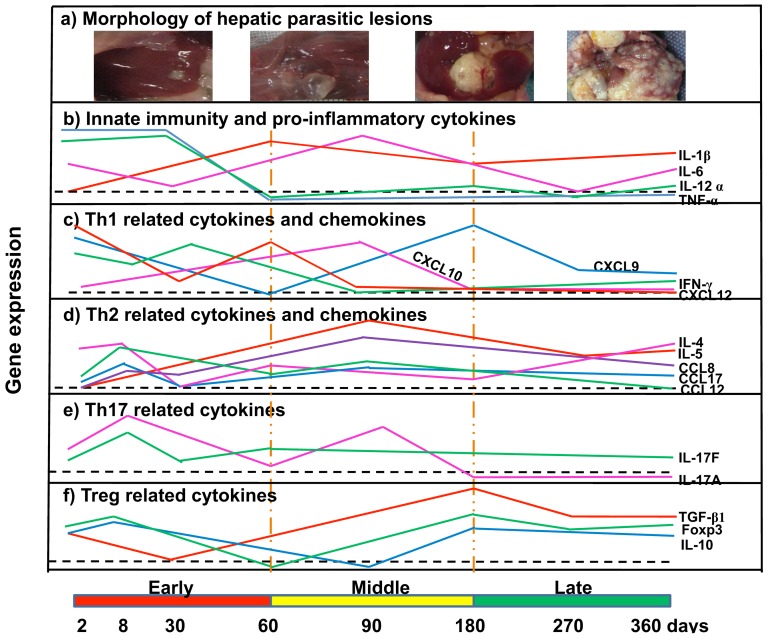
Course of the changes in the morphology of the hepatic lesions (a), gene expression of innate immunity and proinflammtory cytokins (b), Th1 related cytokines and chemokines (c), Th1 related cytokines and chemokines (d), Th17 related cytokines (e), Foxp3 and Treg related cytokines (f) during the process of *E. multilocularis*-infection in mice.

**Figure 7:**
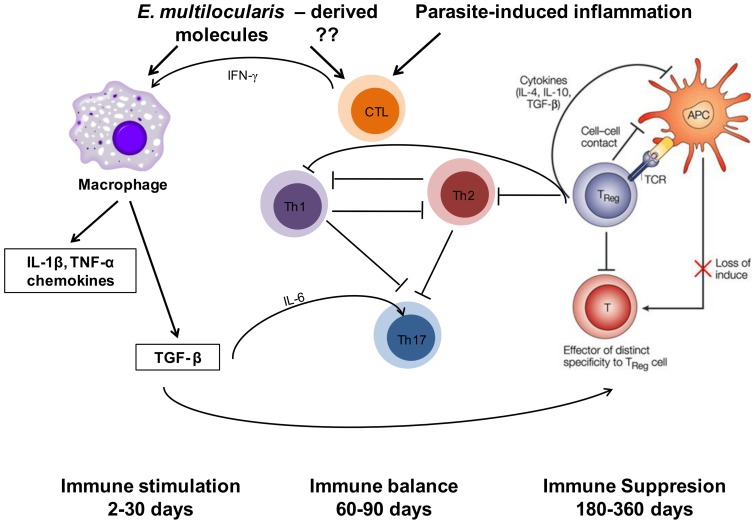
Schematic diagram summarizing the pathways of immune response involved in the host-parasite relationship in *E. multilocularis* infection.

Despite the alleged causative involvement of the granulomatous response in the clinical development of AE and its role in functional imaging of the disease, since it is responsible for the Fluorodeoxyglucose (FDG) uptake in Positron Emission Tomography (PET) [20], a comprehensive picture of the cytokine/chemokine response that occurs in situ, i.e. in the periparasitic granuloma, had never been given. Chemokines and IL-17, which are crucial for immune cell homing, have so far received little attention in *E. multilocularis* infection. In the present longitudinal study of experimental *E. multilocularis* intra-hepatic infection model, we showed for the first time that 1) the mixed Th1/Th2/Treg response and the tri-phasic course of cytokines, suggested by previous studies on spleen cells from *E. multilocularis*-infected mice, was also documented in the periparasitic infiltrate, but nevertheless differed in some aspects, especially the marked and parallel expression of IL-12α and TNF-α but also IL-4 at a very early stage of the parasite/host interactions; 2) IL-17 was involved locally at the beginning of the immune response and remained so all along the course of infection, with a successive expression of different isotypes with possibly different roles; 3) a parallel course of cytokines and their related chemokines was highly in favor of their permanent role to maintain the homing of immune cells at close proximity of the parasitic vesicles; and 4) at least some of the components of the immune response were present in the surrounding liver and were thus involved in a process which was long considered to be a localized “tumor-like” event (Figure 6 and 7).

In the present study, we found that IL-12α and TNF-α were developing in parallel during the different stages of *E. multilocularis* infection. After an initial increase, IL-12α and TNF-α expression decreased dramatically after the 30^th^ day of infection of mice. This fits well to previous findings, which had indicated a protective role against *E. multilocularis* by *in vivo* treatment with recombinant IL-12 in C57BL/6J mice [7], while mice KO for TNF-α [10], as well as patients with AE treated with a TNF-α inhibitor [20], had a faster and more severe course of disease. IL-1β and IL-6 were then showing up, presumably to sustain the inflammatory response, with a ‘mirror’ image of their respective increase all along infection. The initial peak of IL-6 as early as 2 days post-infection may be related to the early activation of the acute phase protein genes in the hepatocytes, disclosed by previous microarray studies [21], [22]. Conversely, the absence of a significant increase of IL-6 at day 270 probably explains why, despite increased levels of haptoglobin, α-1 acid glycoprotein, C3 and C4, and ceruloplasmin in patients with AE, no increase of C-reactive protein (CRP) levels, typically associated with IL-6 stimulation, is usually observed, except in cases complicated by bacterial infection. Secretion of the pro-inflammatory cytokines IL-1β and IL-18 by PBMC of AE patients had been shown to be reduced in response to *E. multilocularis* metacestode vesicles, compared to controls [12]. In our study in mice, although IL-1β was highly expressed at the early and middle stage, it subsequently decreased at the late stage and was not significantly different from control mice at day 180 post-infection and later, a time point which may approximately represent the disease stage of most patients at diagnosis of AE. Such selective dynamics of pro-inflammatory cytokine release may both install and maintain the periparasitic immune infiltrate from the very early stage of infection on, and also limit its activation and thus participate in the tolerance process.

In most previous studies, secretion and expression of cytokines, chemokines, and related factors that govern immune cell-homing to *E. multilocularis* infection site were studied in the peripheral blood of human AE patients [23], and in lymph node or spleen cells of experimentally infected mice [13], [24], [25]; in situ investigations focussing on the periparasitic infiltrate and the adjacent liver tissue are virtually lacking. Early expression of IFN-γ, as previously shown in studies on peripheral lymphocytes, was also confirmed in our longitudinal study of the periparasitic infiltrate; we hypothesize that it was very likely induced by the early expression of IL-12. The apparent decrease in IFN-γ at day 8 may be due either to a technical artefact or, more probably, to a temporary inhibition by IL-4, also markedly expressed at days 2 and 8 p.i.. Sustained IFN-γ expression together with the permanent expression of Th1 chemokines, and its negative correlation with TGF-β1 in the parasitic lesions all along the course of infection, although Th2 and T-reg cytokines are also permanently expressed, suggests that IFN-γ is very important for the persistence of the periparasitic infiltrate by permanent homing of immune cells and/or inhibition of their emigration. The decrease of IL-12 after the early stage of disease could be, at least partly, responsible for the lack of activation of CD8 T-cell or NK cell cytotoxicity despite the presence of IFN-γ [11], [14], [26].

Several concordant observations showed that the PBMCs of AE patients as well as spleen or lymph node cells of experimentally infected mice exhibit a markedly and steadily increasingTh2-oriented response characterized by high levels of IL-4, IL-5 andIL-10 expression [27]. The results from many studies have clearly identified IL-4/IL-5/IL-10 as important regulatory cytokines in parasitic infections, such as infection by *Schistosoma mansoni* in mice [28], [29] and humans [30], *Schistosoma haematobium* [31], *Trichuris muris* [32], and *Trichinella spiralis* [33]. In *E. granulosus* infection, IL-4/IL-5/IL-10 had been found to be predominant in serum samples of infected individuals [34]; furthermore, in the peritoneal cells of experimental mice, i.e. at the site of *E. granulosus* establishment, IFN-γ was secreted first, at day 3, but as early as day 5, a Th2-type response, including IL-4 and IL-13 was stimulated [35]. These results in CE suggest that a Th2-type response does not impair the establishment of *E. granulosus* metacestode, and does not prevent the development of the pericyst, a characteristic of CE pathology, which, conversely to AE, limits the progression of the metacestode [15]. In the parasitic lesions of *E. multilocularis*-infected mice, we observed a biphasic curve of IL-4 mRNA expression, with also a very early peak at 2–8 days. This early peak differed from what is usually reported in *E. multilocularis* infection upon investigation of peripheral lymphocytes stimulated by *E. multilocularis* antigens [5]. The early local expression of IL-4 mRNA might be crucial to prime naive CD4^+^ T cells into differentiated Th2 type cells [35], and to prevent anti-parasite resistance, such as that occurring in most intermediate hosts, including humans. We hypothesize that early IL-4 mRNA expression is likely induced through the activation of innate immunity by specific metabolic components of the metacestode. Such an activation of IL-4 production has actually been described *in vitro* under the influence of *Echinococcus* components, both from *E. multilocularis* [23] and from *E. granulosus* [36]. In the present study, we also found a delayed increase of IL-5 and IL-10 in the middle/late stage of *E. multilocularis* infection. This delayed increase of IL-5 and IL-10 is matching previous observations made by others at the ‘late stage’ of infection, in human AE [37], [38], [39] and are in agreement with the data usually reported from the study of lymphocytes from experimentally infected mice [40]; this combined cytokine profile has been strongly linked to parasite evasion from the host immune response [27], [41].

The discovery of the IL-17 cytokine family has added a new dimension to the balance of inflammation and tolerance during parasite infections. The presence of IL-17-secreting CD4^+^ T (Th17) lymphocytes correlates with severe hepatic pathology in murine schistosomiasis [42]. In our study, IL-17, as detected by a monoclonal antibody directed against the common epitopes of the protein, was present in cells of the periparasitic infiltrate all along the course of infection; however, as far as the expression of mRNA isotypes of the cytokines is concerned, both IL-17A and IL-17F were increased at the early stage of *E. multilocularis* infection, and then decreased at the late stage; they were both positively correlated with CCL12 and CCL17; however, IL-17A exhibited a positive correlation with TNF-α, and appeared lower than even in controls, at the late stage of infection, while IL-17F was also expressed at low levels, but still higher than controls. This may indicate that IL-17A was rather protective but quickly inhibited, while IL-17F was less suppressed with time and may contribute to both protection and pathogenesis, as reported in human AE patients [17].

Chemokines are involved in the homing and persistence of immune cells in inflammatory reactions, especially to infectious agents [43], [44]; they also participate in innate recognition stages of immunity and may help direct Th1 and Th2 cytokine-producing cells during the generation of adaptive immunity [18]. There is also considerable *in vitro* evidence that cytokines further capitalize on these molecules by regulating their expression and secretion and by using them to activate effector cells such as macrophages and fibroblasts [18]. Conversely, specific suppression of certain chemokine production and/or function by *E. multilocularis* metacestode in AE patients may constitute an additional immune escape mechanism [15]. We only measured the mRNA expression of ‘key’ chemokines, directly related to the main cytokine profiles, among the multiple components with chemokine activity. But all measured chemokines were significantly expressed at a given stage of infection. These results confirmed the importance of these compounds to maintain the granulomatous infiltrate at the proximity of the metacestode. The courseof Th1-related chemokines appeared “complementary”; CXCL 9 was more expressed when CXCL10 was less expressed, and vice versa, with a ‘mirror’ image, as previously described for IL-1 and IL-6. This may indicate some balance to ensure lymphocyte homing and persistence in the lesions. Th2-related chemokines were also permanently expressed: expression of CCL12 and CCL17 followed the course of IL-4, and CCL 8 followed the course of IL-5. Such changes in chemokine release may prevent pathogenic inflammation at the late stage. In addition, the microarray technique revealed a hyper-expression of RANTES (CCL5), chemotactic for Th1 cells, eosinophils, and basophils [11]. This finding suggests that this chemokine is also secreted by cells of the granuloma at the early stage (8–30 days) when IL-12, IFN-γ and IL-17 secretions are at their maximum. This should consequently also be explored more in detail in future studies.

The involvement of the adjacent, not directly affected liver tissue in the immune process of *E. multilocularis*/host interaction has received little attention. Recent studies have provided evidence that the adjacent liver was fully involved in the relationship between the parasite and its host; these studies have mostly focused on the proliferation/apoptosis balance [18] and the involvement of the TGF-β/Smad system [19]. Our study confirms that other mediators of the immune reaction and their receptors appear principally expressed in the liver tissue, thus also in areas not directly affected by the parasite and the periparasitic granuloma. In the adjacent periparasitic liver tissue, the expression of the various cytokines/chemokines was selective: not all cytokines/chemokines were expressed in the surrounding liver; some seemed to be specific for the immune cells of the periparasitic infiltrate, e.g. TNF-α, IL-17F and CCL8, which were not expressed at all in the liver. The contribution of the surrounding liver tissue, however, was quite significant for other ones, e.g. IL-12, IFN-γ, IL-4 and IL-17A, at the early stage of infection; CXCL9, IL-4, IL-5, CCL17, at the middle stage; and IL-10 and TGF-β at the late stage of infection. From our study, which was performed on liver samples without cell identification, it is difficult to know if such expression was restricted to cells of the immune response present in the sinusoids/portal spaces after their homing to the liver, or was also present in autochthonous liver cells such as Kupffer cells, stellate cells, or hepatocytes. Precise identification and respective location will require appropriate studies. Among cytokine receptors, only those for IL-1 (IL-R1 like), IL-7, IL-13 (IL-13 Rα1), and IL-17 (IL-17 R) were up-regulated. This indirectly suggests that the liver was affected by at least one pro-inflammatory cytokine (IL-1) and one growth factor (IL-7), and by two types of Th-cytokines (Th2 and Th17). However, absence of up-regulation of IL-6 and TGF-β receptors in hepatic cells is puzzling and has to be further confirmed using other techniques in the same model.

## Materials and Methods

### Ethics Statement

The animal study was performed in strict accordance with the recommendations in the Guide for the Care and Use of Laboratory Animals. The protocol was approved by the Animal Care and Use Committee and the Ethical Committee of First Affiliated Hospital of Xinjiang Medical University (20081205-2). All surgery was performed under sodium pentobarbital anesthesia, and every effort was made to minimize suffering.

### Mice and experimental design

Pathogen-free female BALB/c mice (8–10-week old) purchased from the Animal Center of Xinjiang Medical University (accredited by the ALLLAC) were housed in cages with a 12-h light/dark cycle and provided with conventional rodent chow and water ad libitum. All animals received human care in compliance with the Medical Research Center's guidelines, and animal procedures were approved by the Animal Care and Use Committee and the Ethical Committee of First Affiliated Hospital of Xinjiang Medical University. *Echinococcus multilocularis* (*E. multilocularis*) metacestodes were obtained from intraperitoneal lesions maintained in *Meriones unguiculatus*, and 0.1 mL of pooled lesion suspension was injected into the anterior liver lobe of mice to be experimentally infected. For each autopsy time-point, eight experimentally infected mice were used in the *E. multilocularis* group (n = 8) and compared with five control mice (n = 5), which received an intra-hepatic injection of 0.1 mL sterile saline solution into the anterior liver lobe using the same surgical procedure. Mice were killed at 2 and 8 days p.i., and subsequently at 1, 2, 3, 6, 9 and 12 months p.i., respectively.

### Tissue sampling of the parasitic lesion and surrounding granuloma, and of adjacent non-affected (periparasitic) liver tissue; and histological examination

In *E. multilocularis* infected mice, liver samples were taken both from (1) the parasitic lesion (including liver tissue directly adjacent by 1 mm to the macroscopically visible parasitic lesion, subsequently designated as “parasitic lesion tissue”) for qRT-PCR, histopathology and immunohistochemistry [17]; and from (2) the liver tissue relatively close to the lesion (subsequently designated as “periparasitic liver tissue”), i.e. starting 2 mm from the macroscopic changes due to the metacestode/granuloma lesion, thus avoiding gross contamination of liver tissue by parasitic *E.multilocularis* tissue/cells and correspondingly involved infiltrating host immune cells, for both qRT-PCR and microarray analyses. Tissue fragments were directly deep-frozen in liquid nitrogen. Control samples were taken from the same (anterior) liver lobe from non-infected control mice.

### RNA extraction and cDNA synthesis

‘Lesion’ and ‘periparasitic liver’ tissue samples of each mouse were processed and analyzed separately. Approximately 50 mm^3^ –sized tissue samples from *E. multilocularis* infected mice or same size liver tissue samples from control mice were used to extract total RNA using TRIzol reagent (Invitrogen, Gaithersburg, MD, USA). The quality of RNA was confirmed by formaldehyde agarose gel electrophoresis, and the concentration of RNA was determined by reading the absorbance at 260/280 nm.

cDNA was synthesized from 1 µg of RNA in the presence of ribonuclease inhibitor (Promega, Shanghai, China), dNTPs, Oligo(dT) 18 primers, and RevertAid™ M-Mulv reverse transcriptase in a total of 25 ìL reaction mix.

### Quantitative real-time RT-PCR

qRT-PCR was run in a thermocycler (iQ5 Bio-Rad, Hercules, CA, USA) with the SYBR Green PCR premix (Qiagen, Hilden, Germany) following the manufacturer's instructions. Thermocycling was performed in a final volume of 20 µL containing 2 µL cDNA and 10 pM of each primer (Table 6). To normalize for gene expression, mRNA expression of the housekeeping gene β-actin was measured in parallel. For every sample, both the housekeeping and the target genes were amplified in triplicate using the following cycle scheme: after initial denaturation of the samples at 95°C for 1 min, 40 cycles of 95°C for 5 s and 60°C (or other) for 30 s were performed. Fluorescence was measured in every cycle, and a melting curve was analyzed after the PCR by increasing the temperature from 55 to 95°C (0.5°C increments). A defined single peak was obtained for all amplicons, confirming the specificity of the amplification.

**Table 6:** Primers and cycling parameters of qRT-PCR.

Gene	Gene bank accession	Primer Sequences	Annealing temperature	Expected Size
β-actin	NM_007393	F:5′-AACTCCATCATGAAGTGTGA-3′	60.0°C	248 bp
		R:5′-ACTCCTGCTTGCTGATCCAC-3′		
TNF-α	NM_013693.2	F: 5′- TATGGCCCAGACCCTCACA-3′	60.0°C	199 bp
		R: 5′-GGAGTAGACAAGGTACAACCCATC-3′		
IL-1β	NM_008361.3	F: 5′-ATCTCGCAGCAGCACATC-3	60.0°C	193 bp
		R: 5′-CCAGCAGGTTATCATCATCATC-3′		
IL-6	NM_031168.1	F: 5′-TTCCATCCAGTTGCCTTCTTG-3′	60.0°C	176 bp
		R: 5′-TCATTTCCACGATTTCCCAGAG-3′		
IFN-γ	K00083.1	F: 5′-ACTCAAGTGGCATAGATGTGGAAG-3′	60.0°C	167 bp
		R: 5′-GACGCTTATGTTGTTGCTGATGG-3′		
CXCL9	NM_008599.4	F: 5′-CTGGAGCAGTGTGGAGTTC-3′	60.0°C	167 bp
		R: 5′-CCGTTCTTCAGTGTAGCAATG-3′		
CXCL10	NM_021274.1	F: 5′-CAGAGCCAACGTCAAGCATC-3′	60.0°C	200 bp
		R: 5′-CGTCTTATCCAAGTGGTTTATGGAA-3′		
IL-4	M25892.1	F: 5′-AGTTGTCATCCTGCTCTTC-3′	55.0°C	165 bp
		R: 5′-GTGTTCTTCGTTGCTGTG-3′		
IL-5	NM_010558.1	F: 5′-TGAGGCTTCCTGTCCCTACTCATAA-3′	60.0°C	119 bp
		R: 5′-TTGGAATAGCATTTCCACAGTACCC-3′		
CCL8	NM_021443.3	F: 5′-CTTTGCCTGCTGCTCATAG-3′	60.0°C	150 bp
		R: 5′-GCACTGGATATTGTTGATTCTC-3′		
CCL12	NM_011331.2	F: 5′-GCTACCACCATCAGTCCTC-3′	60.0°C	135 bp
		R: 5′-CTGGCTGCTTGTGATTCTC-3′		
CCL17	NM_011332.3	F: 5′-TCAGTGGAGTGTTCCAGGGATG-3′	60.0°C	151 bp
		F: 5′-GGCGTCTCCAAATGCCTCA-3′		
IL-17A	NM_010552.3	F: 5′-GTGTCTCTGATGCTGTTG-3′	60.0°C	193 bp
		R: 5′-AACGGTTGAGGTAGTCTG-3′		
IL-17F	NM_145856.2	F: 5′-GTCGCCATTCAGCAAGAAAT-3′	60.0°C	148 bp
		R: 5′-CAGCCAACTTTTAGGAGCATCT-3′		
Foxp3	NM_054039.1	F: 5′-GAGAGGCAGAGGACACTCAATG-3′	60.0°C	108 bp
		R: 5′-GCTCAGGTTGTGGCGGATG-3′		
TGF-β 1	NM_011577	F: 5′- GTGTGGAGCAACATGTGGAACTCTA-3′	60.0°C	143 bp
		R: 5′-TTGGTTCAGCCACTGCCGTA-3′		
IL-10	NM_010548.2	F: 5′- GCCAGAGCCACATGCTCCTA-3′	60.0°C	145 bp
		R:5′-GATAAGGCTTGGCAACCCAAGTAA-3′		

### Microarray data analyses and annotation of gene function

RNA extracts from 3 infected and 3 control mice were selected for array hybridization, corresponding to 30 days, 60 days, 90 days and 180 days after infection. Total RNA was purified with NucleospinH RNA Clean-up Kit (Macherey-Nagel, Germany) and each purified RNA sample isolated from an individual sample was run on a single microarray. All microarray procedures were done according to a previously described procedure [27].

The original microarray data have been uploaded to Gene Expression Omnibus (GEO) website: http://www.ncbi.nlm.nih.gov/geo/index.cgi (accession number: GSE24376). All data is MIAME compliant.

### Immunohistochemical analyses

Immunohistochemistry was performed on formalin-fixed, paraffin-embedded tissue: 4 µm tissue sections were de-paraffinized in xylene and rehydrated in gradual dilutions of ethanol. Endogenous peroxidase was blocked with 3% hydrogen peroxide. To increase staining, sections were pre-treated by microwave heating for 15 min in antigen unmasking solution (pH 6.8, 0.1 M citrate buffer, Zhongshan Jinqiao Biology Corporation, Beijing). To block non-specific background, the sections were incubated with non-immune goat serum for 30 min. Sections were then incubated overnight at 4°C with the primary antibody diluted in pH 7.3 phosphate-buffered saline (PBS) (IL-17 1∶100 (Santa Cruz Corporation, CA, USA). After 3 washes in PBS, the sections were subsequently incubated with horseradish peroxidase conjugated host-specific secondary antibodies and 3,3′-diaminobenzidine was used as chromogen. Sections were counterstained with hematoxylin for 5 min, dehydrated, and covered with slips. For all samples, negative controls consisted of substitution of the isotype-matched primary antibody with PBS.

### Expression of the data and statistical analysis

Immunostaining for IL-17 was semi-quantified by calculating “expression scores” that consider both staining intensity and the percentage of cells stained at a specific range of intensities. A score of zero indicated the percentage of positive cells <5%, 1+ = 5–25%, 2+ = 25–50%, 3+ = 50–75%, 4+>75%. The staining intensity of each specimen was judged relative to the intensity of a control slide including an adjacent section stained with an irrelevant negative control antibody that was matched by species and isotype to the specimen. Staining of the section labelled with the negative reagent control was considered as background. A score of zero indicated no staining relative to background, 1+ = weak staining, 2+ = moderate staining, and 3+ = strong staining. According to standard pathology practices, staining intensity was reported at the highest level of intensity observed in all tissue elements, except the distinctive tissue element for which an expanded scoring scheme was reported. The “expression scores” were calculated by multiplying the percentage of positive cells (0–4) and the staining intensity scores (0–3). For example: for a specimen with 30% of positive cells(3+), and a moderate staining intensity (2+), the “expression score” was3×2 = 6. Three pathologists read the sections and established the scores, and they were blinded to each other's results. Cells with a positive immunostaining were counted in five random visual fields of 0.95 square mm each, at initial magnification: ×20, for each sample.

All the data were analysed by SPSS 17.0. mRNA expression of the various cytokines, chemokines, and other components of the immune response of *E. multilocularis* infected mice were compared to the results obtained on the liver samples taken from control mice in the sham-infected liver lobe at the same time point. The results were presented as means ± SD. One-way ANOVA and Student's *t*-test were used to compare the differences between groups, and Spearman's rho was used to analyse the correlation coefficients. *P*<0.05 was considered to indicate statistical significance.
